# The coevolutionary dynamics of cryptic female choice

**DOI:** 10.1093/evlett/qrad025

**Published:** 2023-06-07

**Authors:** Matthew C Kustra, Suzanne H Alonzo

**Affiliations:** Department of Ecology and Evolutionary Biology, University of California, Santa Cruz, CA 95060, United States; Department of Ecology and Evolutionary Biology, University of California, Santa Cruz, CA 95060, United States; Institute of Marine Sciences, University of California, Santa Cruz, California 95060, USA

**Keywords:** individual-based model, sperm competition, postcopulatory sexual selection, reproductive isolation, Fisherian Runaway Selection

## Abstract

In contrast to sexual selection on traits that affect interactions between the sexes before mating, little theoretical research has focused on the coevolution of postmating traits via cryptic female choice (when females bias fertilization toward specific males). We used simulation models to ask (a) whether and, if so, how nondirectional cryptic female choice (female-by-male interactions in fertilization success) causes deviations from models that focus exclusively on male-mediated postmating processes, and (b) how the risk of sperm competition, the strength of cryptic female choice, and tradeoffs between sperm number and sperm traits interact to influence the coevolutionary dynamics between cryptic female choice and sperm traits. We found that incorporating cryptic female choice can result in males investing much less in their ejaculates than predicted by models with sperm competition only. We also found that cryptic female choice resulted in the evolution of genetic correlations between cryptic female choice and sperm traits, even when the strength of cryptic female choice was weak, and the risk of sperm competition was low. This suggests that cryptic female choice may be important even in systems with low multiple mating. These genetic correlations increased with the risk of sperm competition and as the strength of cryptic female choice increased. When the strength of cryptic female choice and risk of sperm competition was high, extreme codivergence of sperm traits and cryptic female choice preference occurred even when the sperm trait traded off with sperm number. We also found that male traits lagged behind the evolution of female traits; this lag decreased with increasing strength of cryptic female choice and risk of sperm competition. Overall, our results suggest that cryptic female choice deserves more attention theoretically and may be driving trait evolution in ways just beginning to be explored.

## Introduction

Sexual selection can drive the evolution of complex traits and result in trait divergence between populations and species (Coyne et al., 2004; Irwin, 2020; Lande, 1981; Mead & Arnold, 2004; Mendelson et al., 2014; Servedio & Boughman, 2017). Changes in the preferences of one sex can impose selection on traits in the other sex, leading to codivergence of these traits among isolated populations (Lande, 1981; Mead & Arnold, 2004; Servedio & Boughman, 2017). Despite numerous theoretical models on intersexual selection (Mead & Arnold, 2004; Servedio & Boughman, 2017; Turelli et al., 2001), almost all theory has focused on premating sexual selection (but see, Lorch & Servedio, 2007; Rushworth et al., 2022). In contrast, little is known about the coevolution between female and male traits shaped by postmating sexual selection (i.e., selection on traits affecting fertilization after mating; Howard et al., 2009; Parker, 1970; Shuker & Kvarnemo, 2021). Thus, theory that explicitly considers postmating intersexual selection is needed to understand these coevolutionary dynamics.

Postmating sexual selection occurs in two nonmutually exclusive forms: sperm competition, when sperm from two or more individuals compete for the fertilization of the same ova (Parker, 1970), and cryptic female choice, when females bias fertilization towards a specific male or sperm trait (e.g., sperm size; Firman et al., 2017; Thornhill, 1983). Cryptic female choice is often mediated by female reproductive physiology (Beirão et al., 2015; Firman et al., 2017; Gasparini et al., 2020; Higginson et al., 2012; Miller & Pitnick, 2002; Lüpold et al., 2016; Lüpold & Pitnick, 2018; Pitnick et al., 2003, 2020; Poli et al., 2019; Rosengrave et al., 2008). Cryptic female choice can either be directional, when all females share the same preference, or nondirectional, when preferences differ among females (Birkhead & Pizzari, 2002; Firman et al., 2017). Nondirectional cryptic female choice results in female-by-male interactions in fertilization success and has been demonstrated in a wide variety of taxa (Bjork et al., 2007; Clark et al., 1999; Devigili et al., 2018; Firman et al., 2017; Fitzpatrick & Lüpold, 2014; Lüpold et al., 2020; Oliver & Evans, 2014; Pitnick et al., 2020; Poli et al., 2019; Rosengrave et al., 2008; Urbach et al., 2005). These female-by-male interactions may be a mechanism for the rapid evolution of sperm, which are the most diverse cell type across taxa, and also show rapid divergence between populations (Hogner et al., 2013; Lüpold & Pitnick, 2018; Manier & Palumbi, 2008; Pitnick et al., 2003, 2009a). Furthermore, there is evidence for the codivergence of female reproductive tract morphology and sperm morphology in various taxa (Briskie et al., 1997; Higginson et al., 2012; Pitnick et al., 2009b, 2020), suggesting cryptic female choice may play a major role in generating such diversity.

One hypothesized mechanism for the coevolution of premating female preferences and male traits is the Fisher Process (Fisher, 1930; Henshaw & Jones, 2020; Kirkpatrick, 1982; Lande, 1981). In the Fisher Process, male traits evolve due to higher mating success. Female preferences evolve from indirect selection arising from genetic correlations between preferences and traits because females mate with males that carry traits associated with their preference. Although a similar process could occur in postmating sexual selection (e.g., sexy sperm hypothesis; Keller & Reeve, 1995; Yasui, 1997), there exists no model that explicitly explores this. Because most premating sexual selection models assume that females are monogamous, the factors that postmating sexual selection empiricists study (e.g., degree of polyandry) are not explored. Additionally, genetic correlations may be harder to establish due to greater stochasticity in the postmating coevolutionary process resulting from (a) selection among males only occurring when multiple mating happens and (b) fertilization not being a “winner-take-all situation” as there will often be mixed paternity in a brood (Bocedi & Reid, 2015; Cramer et al., 2023). Thus, we need theory that explicitly considers postmating sexual selection.

The focus of most postmating sexual selection theory is on intrasexual selection (exclusive sperm competition), not intersexual selection (cryptic female choice; Parker & Pizzari, 2010; Ah-King, 2022; but see Ball & Parker, 2003; Cramer et al., 2023; Lorch & Servedio, 2007). Moreover, most postmating sexual selection theory takes a game theory approach focused on strategic ejaculate allocation (Parker & Pizzari, 2010). While powerful for predicting some evolutionary outcomes, game theory does not address the underlying genetic correlations needed for coevolution (Kirkpatrick, 1982; Kuijper et al., 2012; Lande, 1981). The evolution of genetic correlations between postmating sexually-selected traits and preferences (e.g., sperm length and female reproductive tract length; Miller & Pitnick, 2002) could be hindered by selection simultaneously acting on sperm number, especially if tradeoffs exist. Previous models have explored this tradeoff (Immler et al., 2011; Parker, 1993; Parker et al., 2010), but did not explicitly consider cryptic female choice.

We first tested if nondirectional cryptic female choice causes deviations in predicted ejaculate investment from models that exclusively focus on male-mediated processes. We did this by contrasting a “traditional” game theory model of sperm competition to a genetically explicit individual-based model that either did or did not incorporate cryptic female choice with sperm competition. We then asked how the risk of sperm competition, preference strength, and a tradeoff between sperm number and sperm trait interact to influence postmating coevolutionary dynamics. Specifically, we looked at the magnitude of these genetic correlations between the cryptic female choice trait and the sperm trait, and whether these genetic correlations resulted in trait codivergence across a suite of scenarios.

## Methods

Below, we first describe our analytical model of exclusive sperm competition. We then describe the basic structure of our genetically explicit individual-based model (IBM). Next, we present equations representing how selection acts on male ejaculate traits and explain the details of our simulations and the analyses we performed (see Table 1 for a summary).

**Table 1.** Parameter, variable, and function definitions and corresponding values used in the models.

	Symbol	Definition	Values/equations
Parameters			
	*N*	Population size (IBM)	10,000
	*μ*	Mutation rate (IBM)	0.005
	*Q*	Probability that a female will mate with more than one male (Both)	0.25; 0.5; 0.75; 1
	*α* ^a^	Shape parameter for post- and premating tradeoff (Both)	1/20; 1/1,000
	*β* ^a^	Scale parameter for post- and premating tradeoff (Both)	50; 2,500
	*ω*	Width of optimality function which determines strength of selection; lower values result in stronger selection (IBM)	50; 12.5; 1
	*C*	Ejaculate depletion rate (IBM)	−0.2
Variables	*x*	Ejaculate investment (AM)	Evolves
	*m*	Sperm trait (IBM)	Evolves
	*f*	Cryptic female choice trait (IBM)	Evolves
	*s*	Sperm number (IBM)	Evolves
Functions	*n*_*r*_ *(x*_*m*_ *,x*_*e*_)	Expected mating success of mutant relative to a male at equilibrium (AM)	Equation 2
	*v(x*_*m*_ *,x*_*e*_)	Expected fertilization success of mutant relative to a male at equilibrium (AM)	Equation 3
	*W(x*_*m*_ *,x*_*e*_)	Fitness of a mutant relative to a male at equilibrium (AM)	Equation 4
	*P(z*_*i*_)	Probability of male z_i_ being selected for mating (IBM)	Equations 7 and 8
	*ψ*(z**_*i*_ *,z*_*j*_)	Probability that male z_i_ fertilizes an egg given male competitor z_j_ (IBM)	Equation 9

*Note.* IBM = individual-based model; AM = analytical model; Both = used in both models.

^a^The first value is for no tradeoff between *s* and *m*; the second value is with a trade-off between *s* and *m*. Values were changed to keep the same scale for total investment.

### Analytical model of sperm competition

We developed an analytically tractable game theory model based on Parker (1998) to compare to our IBMs. We assumed a tradeoff between postmating and premating traits. Specifically, we assumed that the mating success probability (*n*) of a male with ejaculate allocation (*x*_*i*_) would decrease as a sigmoidal function with increasing *x*_*i*_ such that *α* is a shape parameter and *β* is the inflection point of the sigmoidal shape,


$$\begin{array}{l} \begin{array}{l} n(x_{i})=1-\frac{1}{1+e^{-\alpha \left(x_{i}-\beta \right)}}. \end{array} \end{array}$$ (1)

We chose a sigmoidal relationship rather than the inverse relationship used in Parker (1998) because we believe that it is more realistic to assume that there should be diminishing returns in *n* when decreasing *x*_*i*_ after a certain point.

The relative expected mating success (*n*_*r*_) for a mutant with ejaculate allocation (*x*_*m*_) to a male with equilibrium ejaculate allocation (*x*_*e*_) is,


$$\begin{array}{l} \begin{array}{l} n_{r}(x_{m},x_{e})=\frac{1-\frac{1}{1+e^{-\alpha \left(x_{m}-\beta \right)}}}{1-\frac{1}{1+e^{-\alpha \left(x_{e}-\beta \right)}}}. \end{array} \end{array}$$ (2)

The expected fertilization success (*v*) of mutant with ejaculate allocation (*x*_*m*_) relative to the male with equilibrium ejaculate allocation (*x*_*e*_) is,


$$\begin{array}{l} \begin{array}{l} v(x_{m},x_{e})=(1-q)+2q\frac{x_{m}}{x_{e}+x_{m}}. \end{array} \end{array}$$ (3)

Where *q* is the risk of sperm competition (probability that a female mates with more than one male). Assuming that females do not differ in fecundity, the fitness of a mutant relative to a male at equilibrium (*W*(*x*_*m*_*,x*_*e*_)) is the product of *n*_*r*_ and *v*,


$$\begin{array}{l} \begin{array}{l} W(x_{m},x_{e})=n_{r}(x_{m},x_{e})v(x_{m},x_{e}). \end{array} \end{array}$$ (4)

To find the evolutionary stable strategy (ESS) ejaculate allocation equation, we found when the derivative of the fitness equation was equal to 0 evaluated at *x*_*m*_*= x*_*e*_,


$$\begin{array}{l} \begin{array}{l} \frac{-\;\alpha x+\frac{q(e^{\alpha (\beta -x)}+1)}{2}}{x(e^{\alpha (\beta -x)}+1)}=0 \end{array} \end{array}$$ (5)

The solution requires the use of the Lambert function, which yields two possible solutions (Lehtonen, 2016):


$$\begin{array}{l} \begin{array}{l} \frac{\frac{q}{2}+LambertW\left(\frac{qe^{\alpha \beta -\frac{q}{2}}}{2}\right)}{\alpha },\frac{\frac{q}{2}+LambertW\left(\frac{-qe^{\alpha \beta -\frac{q}{2}}}{2}\right)}{\alpha }. \end{array} \end{array}$$ (6)

We only use the first solution because the second does not yield positive values for valid values of *q* (i.e., between 0 and 1). Ejaculate allocation increases monotonically with risk of sperm competition with the shape of this curve depending on *α* and *β* (Supplementary Figure S1; SI web app).

### General IBM description

Individuals in this model were diploid and had three positive, continuous traits with sex-limited expression: sperm number (*s*), sperm trait (*m*), and cryptic female choice trait (*f*; Supplementary Figure S2). We performed simulations for both cryptic female choice and sperm competition only (see Supplementary Figure S3 for a flow chart of the model). We ran two sperm competition only models: (a) fixed stabilizing selection on *m* (e.g., optimum sperm velocity given a tradeoff with sperm longevity; Levitan, 2000) and (b) fair raffle where only sperm number (*s*) mattered. Like most models of sperm competition (Parker & Pizzari, 2010), we always assumed that pre- and postmating success traded off with one another. In addition to this pre- and postmating tradeoff, we varied whether there was a tradeoff between *s* and *m* because not all sperm traits will tradeoff with sperm number. For example, we would not necessarily expect a tradeoff between chemical signals/receptors for sperm function and the number of sperm produced by a male (e.g., Fitzpatrick et al., 2020). We refer to these different scenarios as “tradeoff” or “no tradeoff.”

We assumed that females mate with at most two males, with the probability of a second mating occurring being the risk of sperm competition (*q*). If sperm competition (i.e., multiple mating) occurred, a male’s fertilization success depended on how high his sperm number (*s*) was relative to a competitor and how closely his sperm trait (*m*) matched an “optimum” relative to the sperm trait of his competitor. For cryptic female choice simulations, the “optimum” was the cryptic female choice trait (*f*); for simulations of stabilizing selection with sperm competition only, this was a set constant. The strength of preference (*ω*) on *m* determined the selective advantage of differences in the sperm trait value between the two competing males when there was sperm competition. For simplicity we ignore mate order effects. We assumed nonoverlapping generations to keep population size and sex-ratio constant.

### Premating sexual selection

We modeled premating sexual selection like Equation 1. In model runs with a tradeoff between *m* and *s*, the probability that a female mated with male *z*_*i*_ (*P*(*z*_*i*_)) where *n*_*m*_ is the number of males in the population is,


$$\begin{array}{l} \begin{array}{l} P\left(z_{i}\right)=\;\frac{1-\frac{1}{1+e^{-\alpha \left(s_{i}m_{i}-\beta \right)}}}{\overset{n_{m}}{\underset{j=1}{\sum }}\;\;1-\frac{1}{1+e^{-\alpha \left(s_{j}m_{j}-\beta \right)}}}. \end{array} \end{array}$$ (7)

In model runs without a tradeoff between *m* and *s*, the probability that a female mated with male *z*_*i*_ (*P*(*z*_*i*_)) was only dependent on *s*:


$$\begin{array}{l} \begin{array}{l} P(z_{i})=\;\frac{1-\frac{1}{1+e^{-\alpha \left(s_{i}-\beta \right)}}}{\overset{n_{m}}{\underset{j=1}{\sum }}\;\;1-\frac{1}{1+e^{-\alpha \left(s_{j}-\beta \right)}}}. \end{array} \end{array}$$ (8)

For simulations without a tradeoff, we set *α* = 1/20 to make the predicted investment moderately distinct across *q* (Supplementary Figure S1), and *β* = 50 to make the inflection point around starting trait averages. To keep the shape of the tradeoff the same when multiplying *m* and *s*, we set *α* = 1/1,000 and *β =* 2,500 for simulations with a tradeoff. Preliminary analyses varying these parameters did not influence qualitative results.

### Postmating sexual selection

During a competitive mating with male *z*_*i*_ and male *z*_*j*_, male *z*_*i*_'s probability of fertilization *ψ*(*z*_*i*_,*z*_*j*_), was determined by his sperm number (*s*_*i*_) and sperm trait (*m*_*i*_), the competitor male’s sperm number (*s*_*j*_) and sperm trait (*m*_*j*_), the cryptic female choice trait (*f*_*i*_) of the female involved in the mating event, and the strength of preference acting on sperm trait(*ω*; Supplementary Figure S4; SI web app),


$$\begin{array}{l} \begin{array}{l} \psi (z_{i},z_{j})=\;\frac{s_{i}e^{\frac{-{\left(m_{i}-f_{i}\right)}^{2}}{2\omega }}}{s_{i}e^{\frac{-{\left(m_{i}-f_{i}\right)}^{2}}{2\omega }}\;\;+s_{j}e^{\frac{-{\left(m_{j}-f_{i}\right)}^{2}}{2\omega }}}. \end{array} \end{array}$$ (9)

For simulations of stabilizing selection with sperm competition only, *ω* can be thought of as the strength of selection. For simulations of fair raffle, only *s* factored in to determine *ψ.* We assume that females are not under direct selection as fertilization is guaranteed.

To explore the balance between selection on *m* and *s*, we varied the value of *ω* for both cryptic female choice and stabilizing selection simulations. Specifically, we ran simulations when *ω* was 50, which we refer to as weak selection; 12.5, which we refer to as moderate selection; and 1, which we refer to as strong selection (Supplementary Figure S4). To model stabilizing selection with sperm competition only as a comparison to cryptic female choice simulations, we fixed *f* to 50. Preliminary analyses showed that varying this value did not qualitatively change results.

### Model process

We first generated populations with equal sex ratio of size *N*. Each run (population) was randomly initialized with trait values drawn from a normal distribution with mean = 50 and standard deviation (SD) = 5. We did this by assuming a “continuum-of-alleles” (alleles have continuous effects on trait values) and randomly generated two alleles per locus per trait for each individual by drawing from a normal distribution with mean = 1.25 and SD = $\sqrt{0.625}$. We converted any negative numbers to zero, as negative trait values are not biologically meaningful. Each trait was determined by adding each copy of all 20 unlinked loci (Supplementary Figure S2). During each generation, we recorded the mean and standard deviation of traits, the Pearson correlation between the *f* and *m* genetic values of individuals, and the multivariate selection coefficients of each trait (Lande & Arnold, 1983; Stinchcombe et al., 2008; see Supplemental Methods). We calculated ejaculate investment (*x* in the analytical model) as *s* for simulations without a tradeoff and *ms* for simulations with a tradeoff.

Each female mated with at least one male and, with probability *q* a second male. Males, since their potential number of matings was unrestricted, were assumed to experience ejaculation depletion with no recovery at a constant rate of *c* after mating:


$$\begin{array}{l} \begin{array}{l} s^{\prime }=\;se^{-c}. \end{array} \end{array}$$ (10)

We assumed *c* = 0.2 for all runs, preliminary analyses showed that this did not influence results. This ejaculate depletion affected only postmating outcomes (i.e., *ψ*) and not premating success (i.e., *P*). Each female then produced a female and male offspring, with each offspring’s paternity determined by randomly selecting one of the two males with probability *ψ* (Equation 9). In a single mating, that single male sired both offspring. We determined the genotype of each offspring by randomly sampling one allele per locus from each parent. During this process, each allele mutated with probability *μ* = 0.005 and with a mutational effect drawn from a normal distribution with mean = 0 and $SD\;=\sqrt{0.00625}\;$. We limited our evolutionary simulations to 30,000 generations because genetic correlations stabilized by generation 20,000 for all parameters. We ran the model for 50 replicates per parameter combination.

### Analysis of deviation and lags

To test how deviated sperm traits were from their optima, we calculated the deviation of average *m* from the value at which fertilization would be highest (assuming equal *s*). We refer to this as the “optimal” sperm trait value in the results. For cryptic female choice, this was calculated using the average *f* of a population, and for stabilizing selection simulations with sperm competition only, this was 50 (preset optimum).

To test for the possibility of evolutionary lags between *f* and *m*, we looked at the deviation between *f* and *m* in the final 2,000 generations of our simulations. We standardized both traits using the mean of a trait across the final 2,000 generations to keep the scale consistent. We then calculated the mean absolute error (MAE) between standardized *f* and *m* across the final 2,000 generations at different generational lag times (from 0 to 300 generations).

We ran all models using Julia v1.6.4 (Bezanson et al., 2017) and performed analyses/made figures in R (R Core Team, 2020) using the “tidyverse” suite of packages (Wickham et al., 2019). We report a summary of parameters, variables, and values used in this model in Table 1. We conducted several sensitivity analyses on the number of loci that determined each trait, the population size, starting averages, and the starting variation (see Supplemental Methods). All results discussed below are from the IBM and were robust to these sensitivity analyses (Supplementary Figures S5–S11; SI web app). Unless stated otherwise, results reported are from the final generation.

## Results

### What factors influence the evolution of cryptic choice trait, sperm trait, and sperm number?

**Figure 1.**
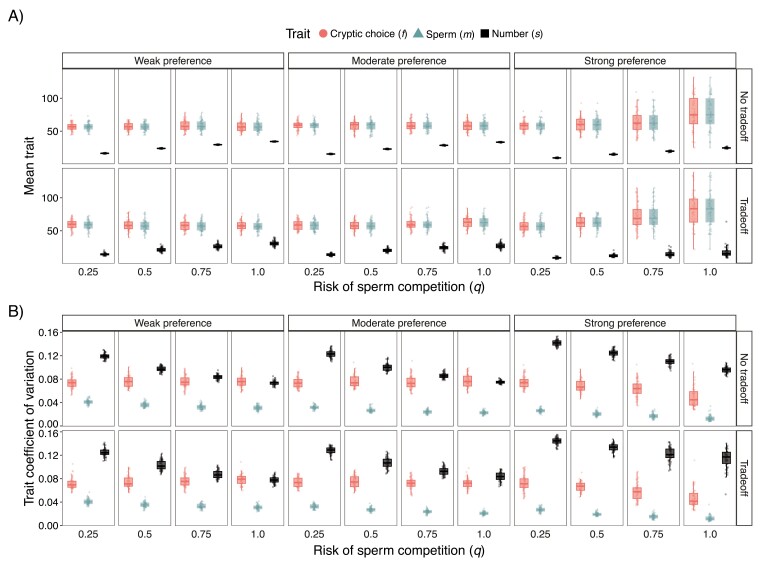
Strong selection and high risk of sperm competition results in higher trait divergence across populations but lower trait variation within populations. (A) Box plots and jittered points of population average of cryptic choice (*f*; left circles), sperm trait (*m*; center triangles), and sperm number (*s*; right squares) at generation 30,000. (B) Box plots and jittered points of within-population coefficient of variation of all traits at generation 30,000. Similar graphs at other parameter combinations can be made on the SI web app.

We first summarize how mean trait values coevolve and diverge across populations, which is essential to understand the potential for reproductive isolation. After 30,000 generations, the presence of a tradeoff between sperm number (*s*) and sperm trait (*m*) did not influence the average trait values of *m* or cryptic choice trait (*f*; Figure 1A). The overall range of average trait values for *f* and *m* increased both with preference strength and risk of sperm competition. There was an interaction such that the risk of sperm competition had a much larger influence when preferences were strong (Figure 1A). We found that *s* increased with the risk of sperm competition as predicted by the analytical model. Unlike *m* and *f*, the range of *s* increased with the risk of sperm competition only when there was a tradeoff.

We then looked at variation maintained within populations after extended coevolution to understand the ability for continual coevolution. We found that the coefficient of variation (CV) was generally highest in *s* followed by *f* and then *m*, with *f* being over double *m* across all scenarios (Figure 1B). The CV of all three traits decreased with the risk of sperm competition (Figure 1B). As the strength of preference increased, the CV of *f* and *m* decreased while *s* increased. This effect was most notable when preferences were strong (Figure 1B). We also found that the range of *s* CV among populations was largest when there was a tradeoff and preferences were strong.

### Strong cryptic female choice results in less overall ejaculate investment

**Figure 2.**
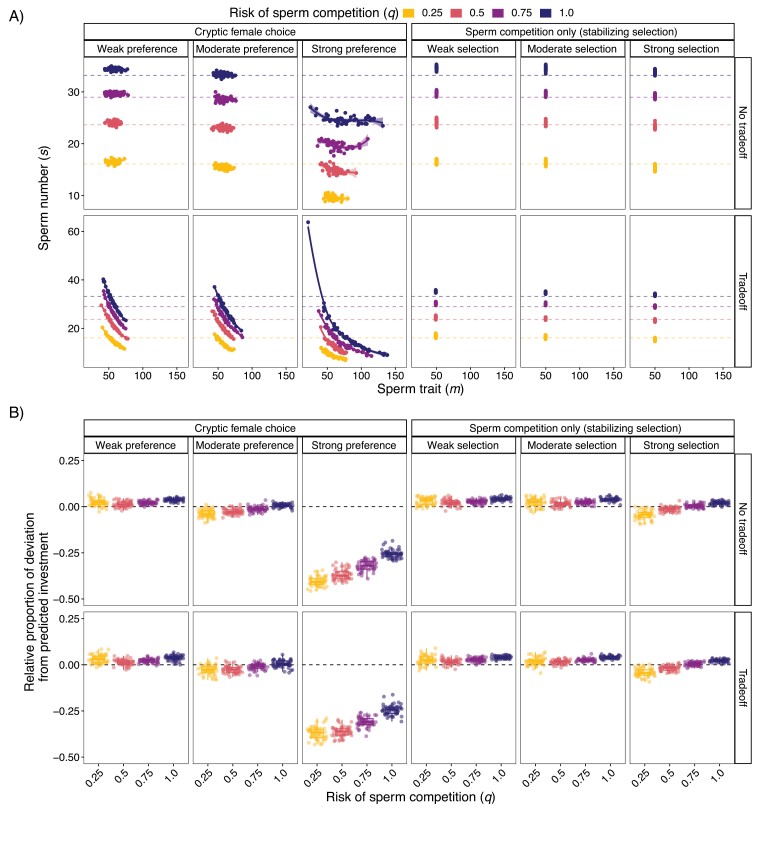
Cryptic female choice can result in less ejaculate investment than models with sperm competition only. (A) Scatter plots of average population sperm trait (*m*) and sperm number (*s*) at generation 30,000 with dashed lines indicating predicted sperm number from the game theory model (*x*_*e*_). Line shown is the best fit local polynomial regression (“LOESS” function) between *m* and *s*. Similar graphs at other parameter combinations can be made on the SI web app. (B) Box plots and jittered points of the population average relative deviation of simulations at generation 30,000 compared to the analytical model’s predicted investment $\left(\frac{simulation\;\;\;investment\text{-}predicted\;\;\;investment\;\;\;}{predicted\;\;\;investment}\right)$. Values at the black dashed line indicate that a simulation exactly matched the game theory model prediction; values above the line indicate more investment than predicted; values below the line represent lower investment than predicted. Simulations of sperm competition only are from simulations with stabilizing selection, fair raffle results are shown in Supplementary Figure S5.

To understand whether cryptic female choice causes deviations from previous theory, we compared the evolutionary stable ejaculate investment from simulations of cryptic female choice to the analytical model with sperm competition only. Our analytical model predicts that sperm number (*s*) should increase with risk of sperm competition. We found that with cryptic female choice when there was a tradeoff between sperm trait (*m*) and *s*, *s* only increased with the risk of sperm competition when comparing populations with similar *m* (Figure 2A). This was unique to cryptic female choice as the simulations with sperm competition only matched the analytical model both qualitatively and quantitively (Figure 2A).

We found that when preference strength was weak, overall investment was larger than predicted by analytical models regardless of the risk of sperm competition and whether a tradeoff was included (Figure 2B). This pattern was also true for simulations with sperm competition only both with and without selection on *m* (Figure 2B; Supplementary Figure S5), indicating that this is a feature of differences in assumptions between the simulations and the analytical model. When preferences were strong, however, it uniquely resulted in much lower ejaculate investment than models with sperm competition only (Figure 2B; Supplementary Figure S5). As the risk of sperm competition increased, overall ejaculate investment became closer to the analytical predictions, but was still ~25% lower.

### Cryptic female choice results in correlated trait evolution even when female preference is weak and risk of sperm competition is low

**Figure 3.**
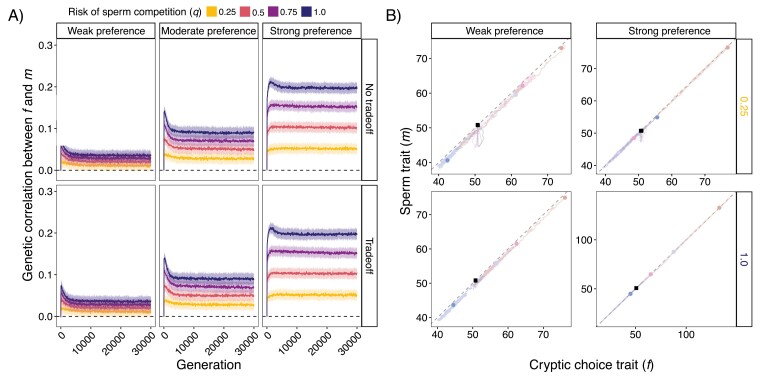
Cryptic female choice results in coevolution even with weak selection and low risks of sperm competition. (A) Genetic correlations between cryptic female choice trait (*f*) and sperm trait (*m*) evolve within the first 200 generations and are maintained due to linkage disequilibrium. Black dashed line is at zero representing no correlation. Lines represent mean and bands represent standard deviation of 50 populations (separate runs) at each parameter combination. (B) When looking across populations, average *f* and *m* are highly correlated. Shown are the highest, lowest, and two random population trajectories of average *f* and *m* when there was a tradeoff and for different preference strengths (weak, strong) and risks of sperm competition (1.0, 0.25). Black dashed line represents a perfect correlation; black square represents starting values; circle dots represent the population ending point after 30,000 generations with different colors representing different populations. Note that the axes differ for the different subpanels. Only every 50 generations are shown due to computer memory constraints when plotting. Similar graphs at other parameter combinations can be made on the SI web app.

We found a positive genetic correlation arose and was maintained between the cryptic female choice trait (*f*) and sperm trait (*m*) across all model scenarios with cryptic female choice (Figure 3). Genetic correlations did not develop for models with sperm competition only (SI web app). Within a few generations, genetic correlations became positive, indicating *f* and *m* loci entered linkage disequilibrium (Figure 3A). This genetic correlation peaked within the first 200 generations, then later declined and stabilized by 10,000 generations (Figure 3A; SI web app). The genetic correlation between *f* and *m* increased with preference strength and the risk of sperm competition. There was an interaction such that the effect that the risk of sperm competition had on genetic correlations increased with preference strength (Figure 3A). These patterns still held when starting *f* was much larger than *m* and there was net directional selection on *m* (Supplementary Figures S6 and S7). The genetic correlations resulted in the codivergence of *f* and *m* (Figure 3B). Populations with a tradeoff that were not under strong preferences drifted along a line slightly below a line of perfect correlation (Figure 3B).

### Cryptic female choice results in a greater deviation from the “optimal” sperm trait predicted by sperm competition only

**Figure 4.**
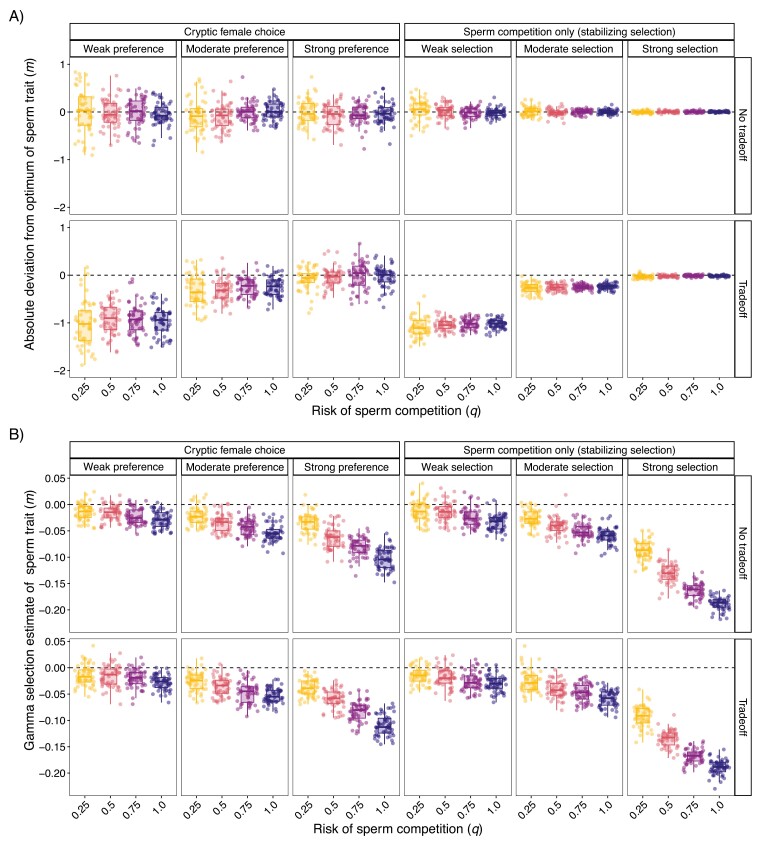
Cryptic female choice results in more deviation from sperm trait optimum than sperm competition only. (A) Box plots and jittered points of simulation deviation from “optimal” sperm trait value (*m;* the value where fertilization is maximized when only considering *m*) at generation 30,000. For cryptic female choice, deviation was calculated by subtracting mean *m* from mean cryptic female choice trait (*f*). For sperm competition only, deviation was calculated by subtracting mean *m* from 50, the optimum set during those runs. Black dashed line indicates zero or no deviation from the optimum. (B) Box plots and jittered points of gamma quadratic selection estimates of *m* after 30,000 generations (Lande & Arnold, 1983). Zero means no quadratic selection (black dashed line), negative values represent stabilizing selection, positive values represent disruptive selection. Coefficient estimates remained stable after 10,000 generations. Similar graphs at other parameter combinations can be made on the SI web.

To understand the degree of trait matching we might see empirically, we tested for deviations between sperm traits and their “optimal” value—where fertilization would be highest when not considering sperm number or mating success. For both cryptic female choice and sperm competition only stabilizing selection simulations, when a tradeoff with sperm number (*s*) was present, the deviation of the sperm trait (*m*) from its optimum was lowest (~1 lower) with weak selection/preference and only slightly lower (~0.25 lower) for moderate selection/preference (Figure 4A). When selection/preference on *m* was strong, *m* did not deviate much from this optimum, indicating selection for *m* overpowered selection on *s*. For simulations with sperm competition only, the range of deviations from the realized optimum (i.e., range of *m*) decreased with increasing selection strength but was not affected by the risk of sperm competition. For cryptic female choice simulations, however, there were sizable deviations from the optimum even with strong preferences (Figure 4A). These deviations were not influenced by the risk of sperm competition.

We tested if differences in deviations were due to differences in the realized strength of selection (gamma quadratic selection coefficient; Lande & Arnold, 1983). We found that gamma became more negative (stronger realized selection) with increasing risk of sperm competition and preference strength (1/*ω*). There was an interaction such that the effect the risk of sperm competition had on gamma increased with the strength of preference (Figure 4B). When preference was weak or moderate, the sperm competition only and cryptic female choice simulations had similar gamma estimates despite cryptic female choice simulations having larger deviations in these scenarios (Figure 4B). However, when selection was strong, simulations with only sperm competition had larger absolute gamma estimates than cryptic female choice simulations.

### Sperm trait evolution lags cryptic choice trait evolution

**Figure 5.**
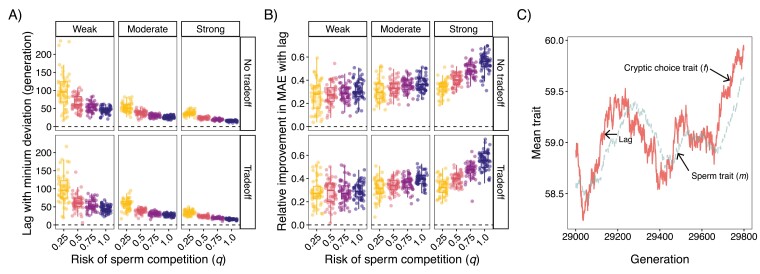
Evolution of sperm trait lags cryptic female choice trait and the length of this lag decreases with strength of cryptic female choice and risk of sperm competition. (A) Box plots and jittered points of generational lag that resulted in minimum mean absolute error (MAE) for different replicates during the last 2,000 generations of the simulation. Dashed horizontal line is at zero indicating no lag best fits the data. (B) Box plots and jittered points of the relative improvement in MAE of incorporating the best fit generational lag versus no lag. Dashed horizontal line is at zero indicating lag did not improve fit. (C) Line plot of an example population showing how the evolution of sperm trait (*m*; dashed) lags the evolution of cryptic choice trait (*f*; solid). The optimal lag in this example population was 49 generations and is when there was no tradeoff, risk of sperm competition was 0.25, and selection was strong.

We next tested if deviations between cryptic choice (*f*) and sperm trait (*m*) could be explained by lags in evolution. We found that regardless of scenario, incorporating an evolutionary lag of *m* improved fit (Figure 5). The length of these generational lags decreased with increasing risk of sperm competition and preference strength (Figure 5A). Furthermore, the relative improvement in deviation between traits increased with risk of sperm competition and preference strength (Figure 5B).

## Discussion

Despite extensive research on sperm competition and the evolution of sperm traits, little is known about the coevolutionary dynamics between sperm traits and female preferences that exert selection on sperm (Lindsay et al., 2019; Parker & Pizzari, 2010). We found that the correlated evolution of cryptic female choice and sperm traits could occur even when there was a tradeoff between sperm trait and number, selection on sperm traits was weak, and the risk of sperm competition was low. We also found that strong cryptic female choice results in males investing less in their ejaculate than predicted by models without cryptic female choice.

Most postmating theory focuses exclusively on male-mediated processes. Thus, it is essential to understand how incorporating cryptic female choice can result in deviations from existing expectations. When selection via nondirectional cryptic female choice was strong, we find males evolved to invest less in their ejaculates than when there was sperm competition only. This counterintuitive result may arise because there is no “best” male trait when the outcome is dependent on female-by-male interactions. Since the sperm trait that maximizes fertilization success depends on female traits, it is likely advantageous to invest more resources toward gaining mating opportunities even when the risk of sperm competition is high. Less investment came at the cost of sperm number since tradeoffs did not limit the extent to which the sperm trait could exaggerate, as the largest sperm trait values evolved under strong selection (Figure 1A). This fits well with empirical work on the *Drosophila* genus, where cryptic female choice is known to occur, with some species producing extremely large but very few sperm (Immler et al., 2011; Lüpold et al., 2016). Although the qualitative prediction that ejaculate investment increaes with the risk of sperm competition remained true for a given preference strength, species likely differ in preference strength. Thus, failing to account for preference strength may cause qualitative predictions to no longer hold. For example, our model predicts that overall investment for a species with strong cryptic female choice and a risk of sperm competition = 1 would have as high of a sperm number as a species with moderate cryptic female choice and risk of sperm competition = 0.5. These results highlight the importance of better characterizing the actual strength of cryptic preferences in different species. Without taking this into account, many previous theoretical predictions (i.e., sperm number increases with risk of sperm competition) may be inaccurate.

Establishing and maintaining a genetic correlation between female preferences and male traits is essential for the premating Fisher Process (Fisher, 1930; Henshaw & Jones, 2020; Kirkpatrick, 1982; Lande, 1981), but under what scenarios can these develop with postmating traits and what factors influence their magnitude? We found that within-population genetic correlations between *m* and *f* increased with preference strength and risk of sperm competition. There was an interaction such that the effect the risk of sperm competition had on genetic correlations increased with preference strength (Figure 3A). Although the magnitude of genetic correlations was generally small (<0.05) when the risk of sperm competition was low with weak preferences, it still resulted in large phenotypic divergence between replicates (Figures 1A and 3B). The resulting phenotypic coevolution of the cryptic female choice trait and the sperm trait predicted by our model is consistent with comparative evolutionary studies documenting the codiversification of the female reproductive tract and sperm morphology (Higginson et al., 2012; Pitnick et al., 2009b, 2020) and female and male genitalia (Evans et al., 2013; Simmons & Fitzpatrick, 2019). Divergence between populations could be aided by female variation being more than twice as large as male variation regardless of modeling scenario. Our results imply that postmating intersexual selection could be an under-appreciated evolutionary force driving reproductive divergence and isolation, even in systems with rare multiple mating. We think it is important for future empirical efforts to look for evidence of cryptic female choice in systems with low to moderate rates of multiple mating.

When designing empirical studies, it is important to know what deviations between coevolving traits we might expect and the biological reasons for these deviations. Logically, we might predict that the amount of deviation between male and female traits important in cryptic female choice will be negatively correlated with the strength of preference and the risk of sperm competition. Our results (Figures 4 and 5), however, predict that deviations between male and female postmating traits are likely to exist and not correlate with preference strength or the risk of sperm competition (Figure 4A). The deviations observed are in part a result of time-lags in male sperm trait tracking a co-evolving female trait and not just a lower realized selection strength than sperm competition only models (Figures 4 and 5). The lower realized selection could be due to variation in female traits (Cramer et al., 2023). Incorporating a generational time lag improved correspondence between male and female traits, especially when preferences were strong and risk of sperm competition was high. To increase the likelihood of detecting cryptic female choice, we suggest using individuals from a wide range of populations for both experimental and comparative work to have sufficient trait variation. Further, the importance of time lags in understanding the coevolution of these traits means that analyzing long-term data sets of population traits will also help with the detection of cryptic female choice.

Our modeling framework provides a strong starting point for future cryptic female choice models and future work should relax some of our assumptions. For simplicity, we only considered a single pair of coevolving traits and sperm number, however, the actual number of traits that may be interacting with one another is much higher (Lüpold et al., 2020; Pitnick et al., 2020; Snook, 2005). Future models could incorporate multiple traits that may be simultaneously under both intra- and inter-sexual selection. We also assumed that preferences and traits were unrelated to fecundity or survival, which impacts premating sexual selection models (Kirkpatrick & Ryan, 1991; Kuijper et al., 2012). Relaxing these assumptions with a good-genes approach could allow positive covariation between premating and postmating success (see Mautz et al., 2013) and potentially allow for the evolution of costly preferences. In our model, we focused on nondirectional cryptic female choice and chose a stabilizing closed preference function to model these female-by-male interactions. Modeling directional cryptic female choice with an open preference function could alter some of the predictions like it does in premating sexual selection models (Jennions & Petrie, 1997; Millan et al., 2020). It would also be interesting to explore sexual conflict (Brennan & Prum, 2015), evolution of negative correlations (Simmons & Kotiaho, 2007), mate order effects (Parker & Pizzari, 2010), the evolution of polyandry (e.g., sexy sperm hypothesis Bocedi & Reid, 2015; Keller & Reeve, 1995; Yasui, 1997), and preference strength. We also assumed that population size and sex ratio were constant; incorporating eco-evo dynamics to relax these assumptions would be an interesting extension. Finally, future work should explicitly explore the degree to which codivergences generated by cryptic female choice can cause reproductive isolation given previous theory showing that premating sexual selection alone can often be ineffective (Irwin, 2020; Servedio & Bürger, 2014). Such a model could allow fitness costs associated with divergence in male and female traits similar to Lorch & Servedio (2007) and Rushworth et al. (2022), which modeled the evolution of conspecific gamete precedence, postmating-prezygotic incompatibilities, and reinforcement.

We demonstrate that incorporating cryptic female choice results in strong deviations from predictions based on models that focused exclusively on male-mediated processes. We also find that strength of selection/preference and the risk of sperm competition often have interactive effects, something that most previous theory and comparative work do not consider. Further, we find that coevolution between female and male traits occurs even with weak cryptic preferences and low rates of multiple mating. Our results highlight the importance of considering cryptic female choice in understanding the evolution of male traits, the need to develop further theory on cryptic female choice, and the importance of conducting more empirical research, especially on the strength of selection arising from cryptic female choice.

## Supplementary material

Supplementary material is available online at *Evolution Letters* and the SI web app can be accessed at: https://mck8dg.shinyapps.io/SI_Evolutionary_dynamics_cryptic_female_choice/.

qrad025_suppl_Supplementary_Material

## Data Availability

Data and code to run the model and make figures are deposited in the Dryad Digital Repository: https://doi.org/10.7291/D1310W (Kustra & Alonzo, 2023).
