# Supplementary material for: The coevolutionary dynamics of cryptic female choice
Source: Evol Lett. 2023 Jun 7;7(4):191–202. doi: 10.1093/evlett/qrad025 (PMC10355280; doi:10.1093/evlett/qrad025)
Supplement: qrad025_suppl_Supplementary_Material [file qrad025_suppl_supplementary_material.pdf]

Online Supplement:

The coevolutionary dynamics of cryptic female choice
https://doi.org/10.1093/evlett/qrad025

Matthew C. Kustra[1] and Suzanne H. Alonzo[1]

[1]Department of Ecology and Evolutionary Biology, University of California, Santa Cruz, California 95060, USA

[*]**Correspondence**: mkustra@ucsc.edu

Supplemental web app:

http://mck8dg.shinyapps.io/SI_Evolutionary_dynamics_cryptic_female_choice/

Supplemental Methods

Calculation of multivariate selection estimates

To understand the realized strength of selection acting on male traits, we performed a multivariate selection analysis on males during each generation using the genotypic value of sperm trait (*m*), sperm number (*s*), and cryptic choice trait (*f*; Lande & Arnold, 1983; Stinchcombe *et al.*, 2008). We included *f* to account for potential correlated selection; excluding *f* in preliminary analyses yielded similar results. We first standardized each trait to a mean of zero and a standard deviation of 1 using the mean value of all males in the population. We then calculated each male's relative fitness (W_r) by dividing the number of offspring sired by the mean number of offspring sired. We then performed a regression analysis to estimate directional selection coefficients (β) and quadratic selection coefficients (γ; Lande & Arnold, 1983; Stinchcombe *et al.*, 2008):

$$W_r = \beta_m m + \beta_s s + \beta_f f + \frac{1}{2}\gamma_{mm} m^2 + \frac{1}{2}\gamma_{ss} s^2 + \frac{1}{2}\gamma_{ff} f^2 + y_{ms} ms + y_{mf} mf.$$

Sensitivity analyses

The results presented in the main text assumed the population size was N =10,000, all traits had starting distributions of mean = 50 and SD = 5, and each trait was controlled by 20 loci. We performed several sensitivity analyses to see if these assumptions had qualitative influences on our results.

To test whether the strength of genetic drift affected our main results, we ran the simulation for smaller populations (N = 1,000), as genetic drift would be more likely to affect predicted patterns than in larger populations with N = 10,000.

Starting conditions and starting variation of traits can affect the coevolution between male and female traits in individual-based models (e.g., Millan *et al.*, 2020), so we ran simulations at every parameter combination at both small trait variation (SD = 2.5) and large trait variation (SD = 10) compared to medium trait variation (SD = 5) for all traits. We additionally ran simulations when starting mean cryptic choice trait (*f*) was ~5 SDs larger than starting mean sperm trait (*m*), 75 vs 50. This allowed us to look at how directional selection could influence our results and/or how large initial deviations between male and female traits influenced our results.

One of our model's key assumptions is that twenty loci control each trait. We ran our model as described above when two loci determined traits to test how this assumption affected our results. To control for the scale of traits, we modified the distributions of mutational effects as well as starting allelic values to maintain approximately the same genetic variation and starting average as the 20-locus model; small: normal (mean = 12.5, SD = 1.25), medium: normal (mean = 12.5, SD = 2.5), and large: normal (mean = 12.5, SD = 5). We also scaled the distribution of mutational effects [normal (mean = 0, SD = 0.25)].

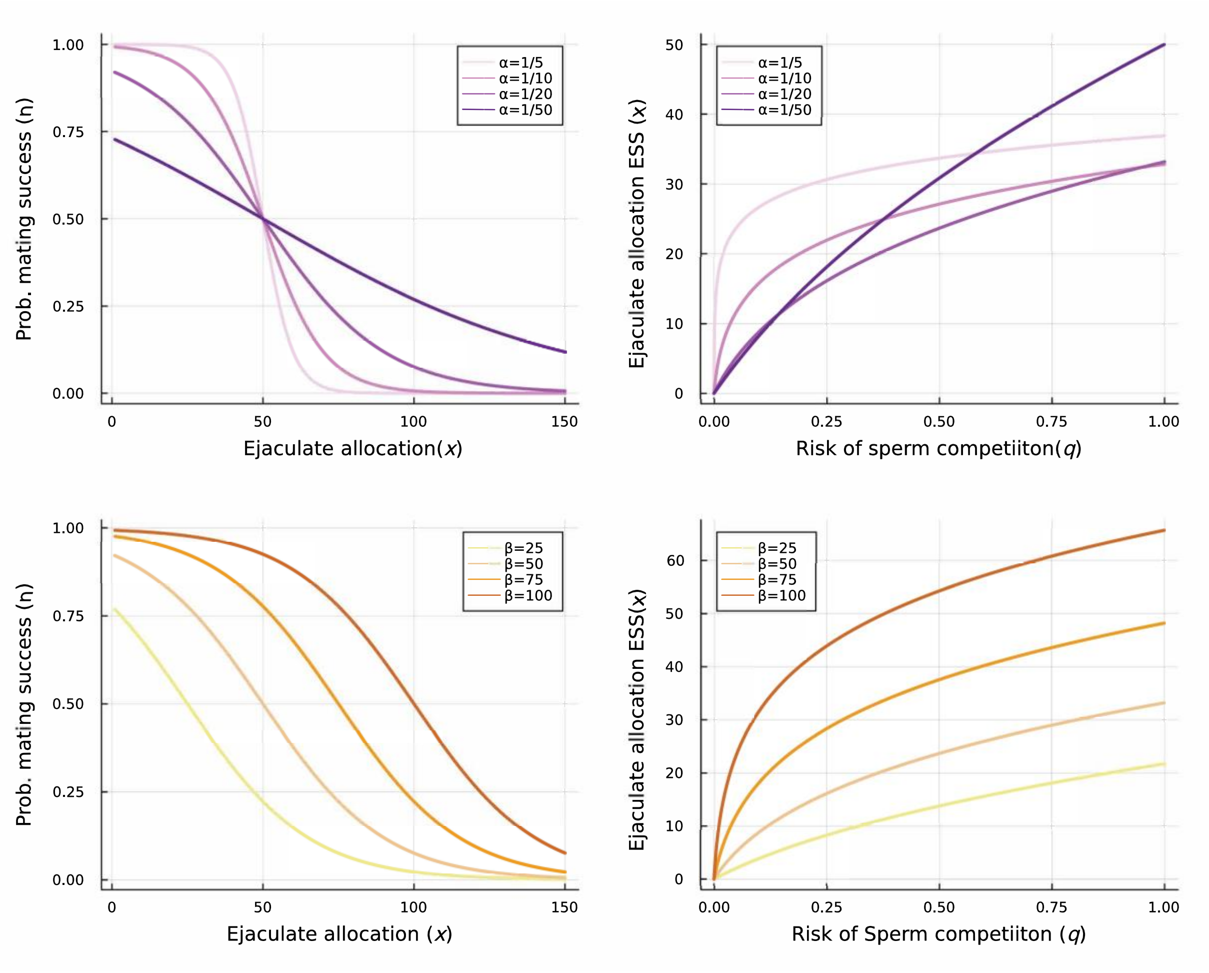

Fig. S1. Demonstration of how model parameters influence the tradeoff between pre- and post-mating success and predicted ejaculate investment of the game theoretic model. Left panels represent how changing α (top left) and β (bottom left) influences the shape of the tradeoff between post- and pre-mating sexual selection (Eq.1). Right panels show how the evolutionary stable strategy (ESS) ejaculate investment (x) varies with risk of sperm competition across different α (top right) and β (bottom right) calculated with Eq.6. Top panels are when $\beta = 50$ and bottom panels are when $\alpha = 1/20$. The SI web app allows one to make similar graphs varying α and β.

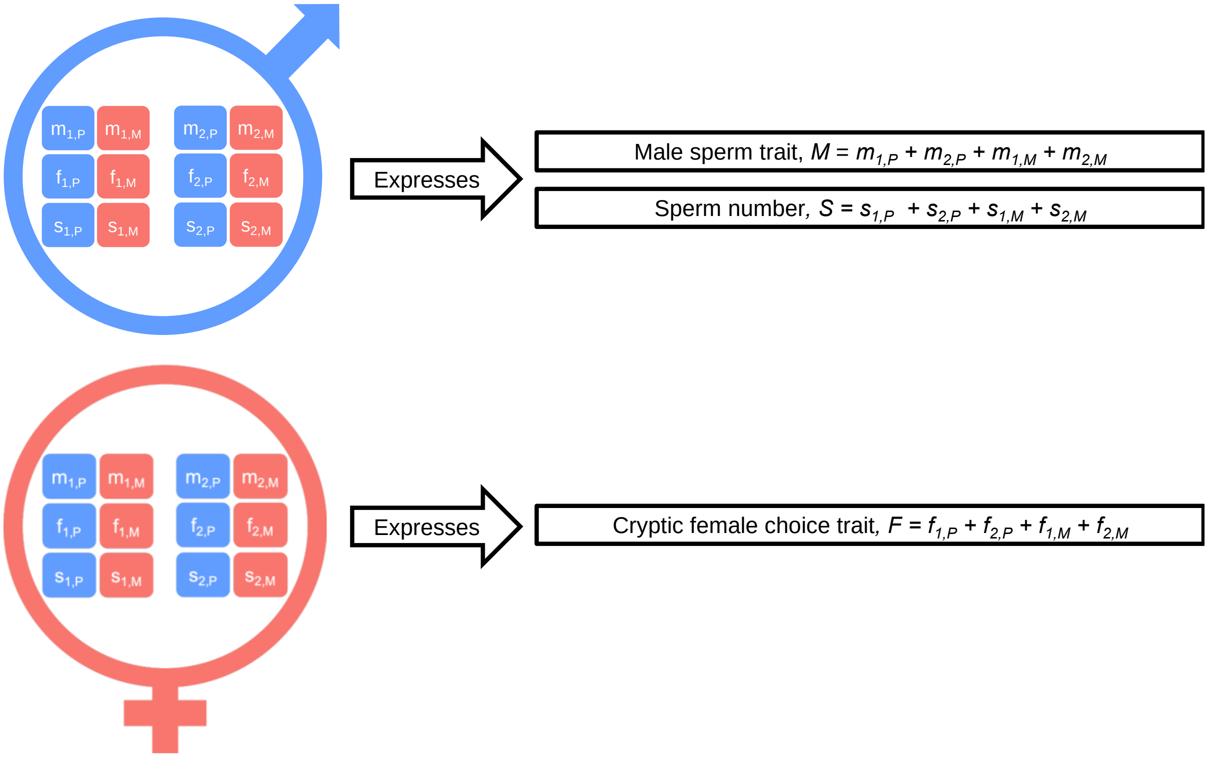

Fig. S2. Diagram of the genetic architecture used and how genotype generates an individual's phenotype in our model for 2 loci runs. *m* = male sperm trait alleles, *f* = cryptic female choice trait alleles, *s* = sperm number. For subscripts, 1 or 2 refers to loci number of a given trait loci; P and M refer to paternally derived (blue) and maternally derived (red) alleles respectively. 20 loci model runs follow the same structure but use 20 loci instead of 2. All traits are continuous and additive across both copies of each loci.

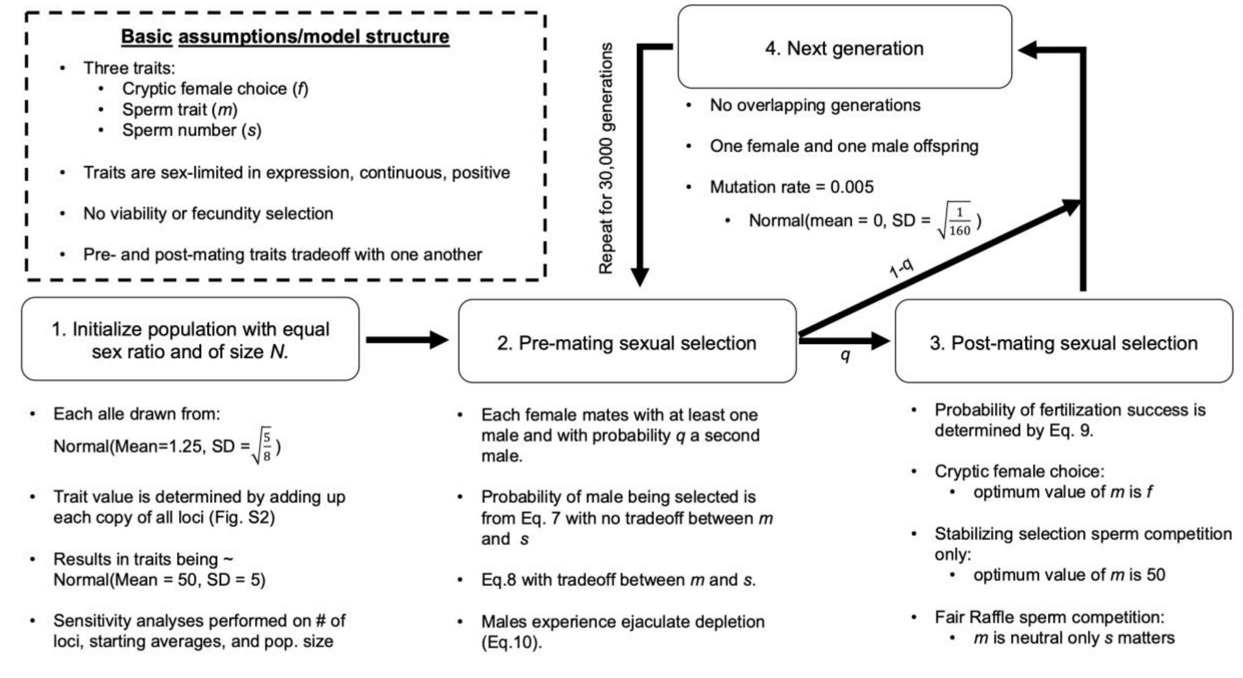

Fig. S3. Flow chart of key steps and assumptions of the individual based model.

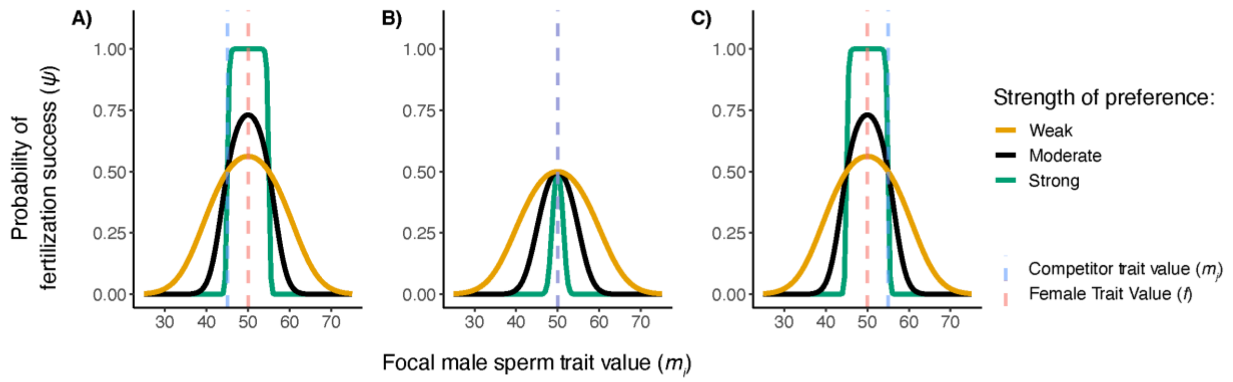

Fig. S4. Graphical representations of Eq.9 showing the probability of fertilization of focal male *i* as a function of the strength of preference (ω), female choice trait value (*f*; red dashed line), sperm trait value of focal male (m_i), and sperm trait value of competitor male (m_j; blue dashed line) when holding sperm number constant. The fertilization advantage of being closer to the female choice trait value increases as preference strength increases (decreasing ω). (A) The competitor male's sperm trait value, 45, is lower than the female choice trait value, 50. (B) The competitor male's sperm trait value, 50, is equal to the female choice trait value, 50. (C) The competitor male's sperm trait value, 55, is greater than the female choice trait value, 50. Weak preference is when $\omega = 50$, moderate preference is when $\omega = 12.5$, and strong preference is when $\omega = 1$. The SI web app allows one to make graphs varying both the cryptic female choice trait, competitor trait values, and both males' sperm number.

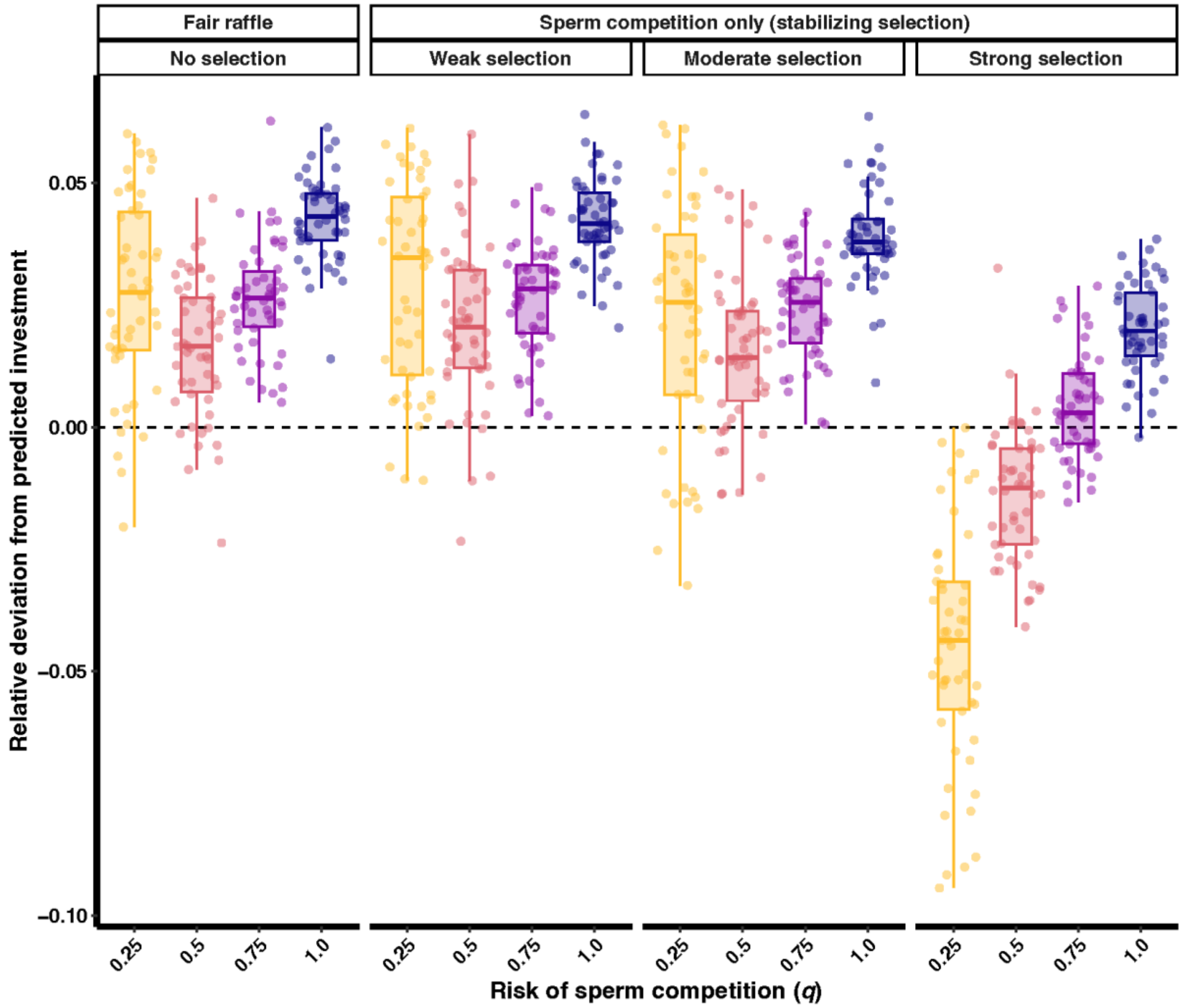

Fig. S5. Box plots and jittered points of the population average relative deviation of simulations at generation 30,000 compared to the analytical model's predicted investment ($\frac{simulation\ investment - predicted\ investment}{predicted\ investment}$) for both fair raffle and stabilizing selection simulations. Values at the black dashed line indicate that a simulation exactly matched the analytical model prediction; values above the line indicate more investment than predicted; values below the line represent lower investment than predicted. Compare to figure 2B in the main text.

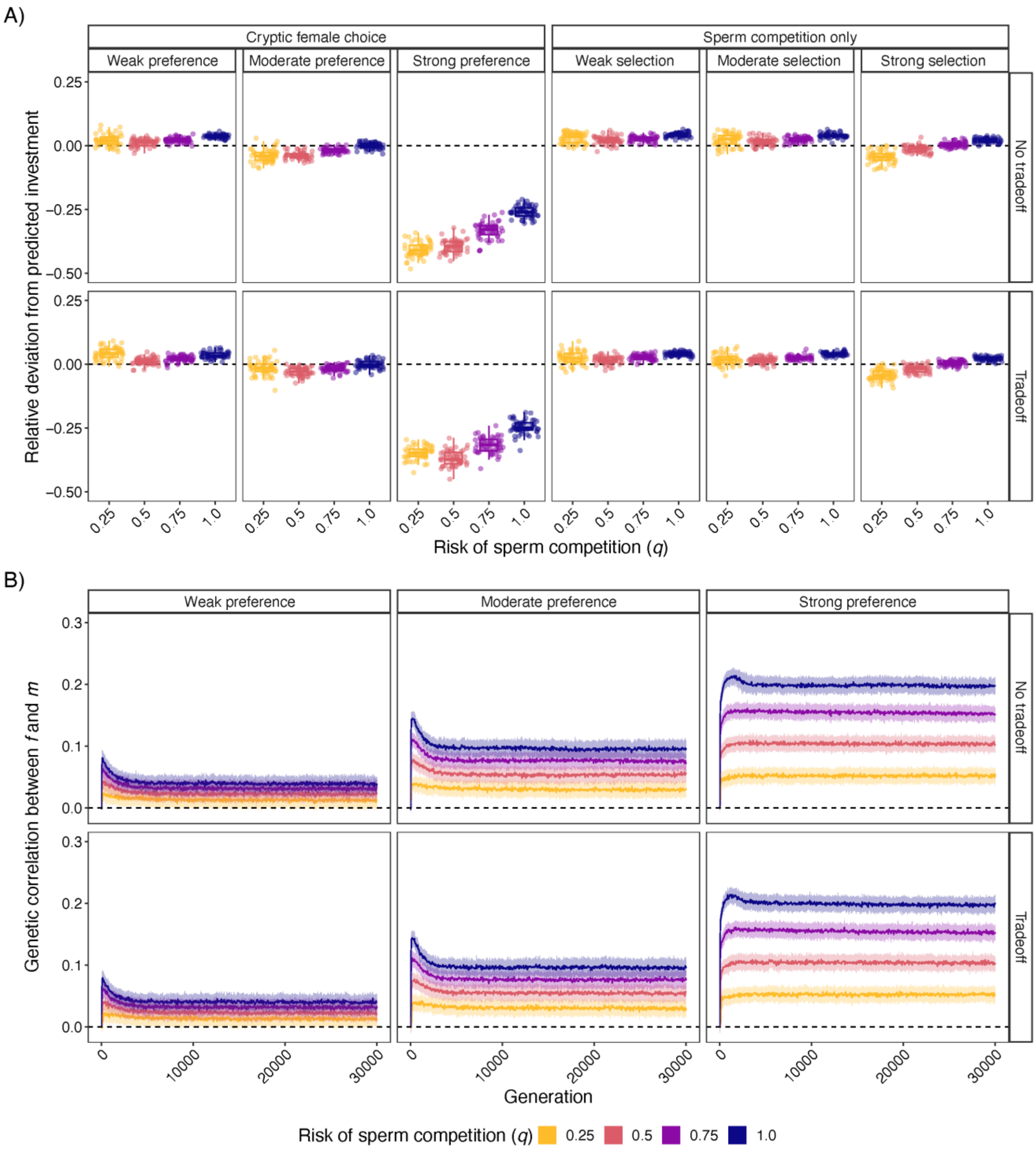

A)
Cryptic female choice
Sperm competition only
Weak preference
Moderate preference
Strong preference
Weak selection
Moderate selection
Strong selection
No tradeoff
Tradeoff
Relative deviation from predicted investment
Risk of sperm competition (q)
B)
Weak preference
Moderate preference
Strong preference
Genetic correlation between f and m
Generation
Risk of sperm competition (q)
0.25
0.5
0.75
1.0

Fig. S6. Making starting cryptic female choice trait (*f*) much larger than sperm trait (*m*) did not influence main results. (A) Box plots and jittered points of the population average relative deviation of simulations at generation 30,000 compared to the analytical model's predicted investment ($\frac{simulation\ investment - predicted\ investment}{predicted\ investment}$). Values at the black dashed line indicate that a simulation exactly matched the game theory model prediction; values above the line indicate more investment than predicted; values below the line represent lower investment than predicted. Compare to main text fig. 2B. (B) Genetic correlations between cryptic female choice trait (*f*) and sperm trait (*m*) evolve within the first 200 generations and are maintained due to linkage disequilibrium. Black dashed line is at zero representing no correlation. Lines represent mean and bands represent standard deviation of 50 populations (separate runs) at each parameter combination. Compare to main text fig. 3A.

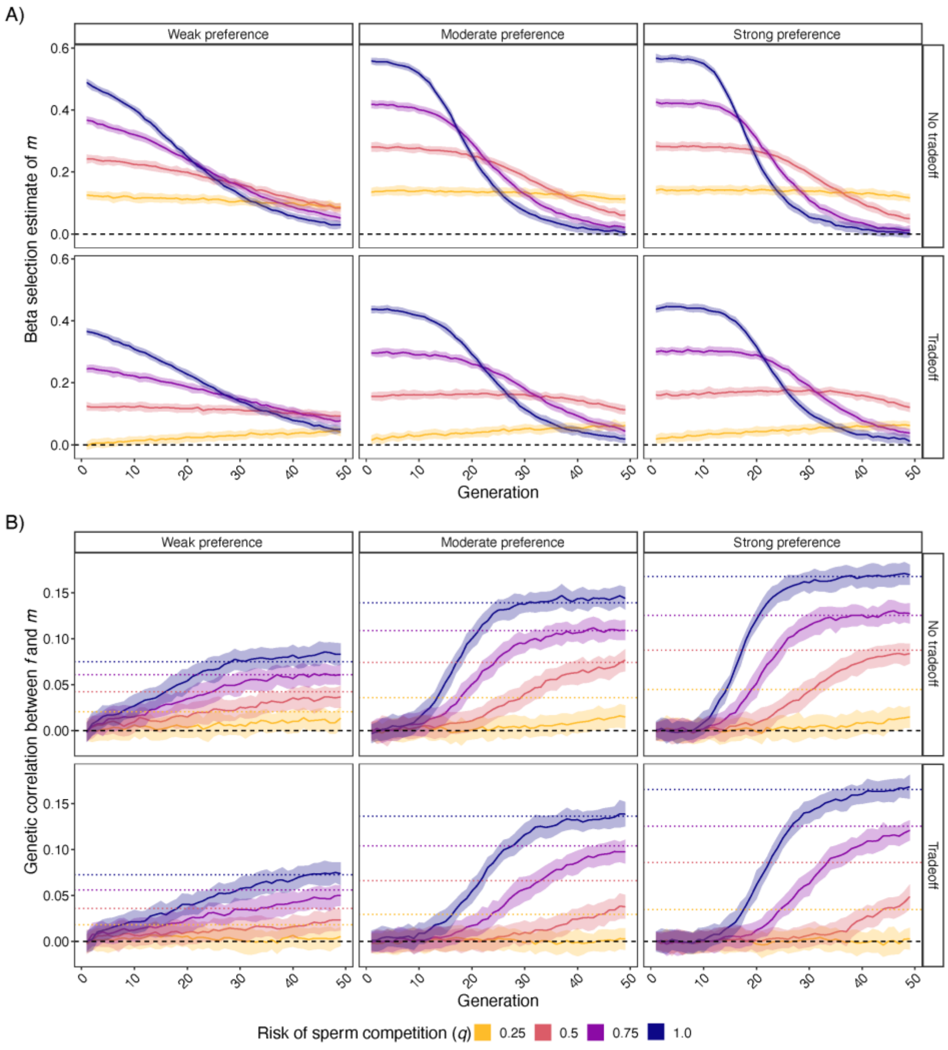

Fig. S7. Genetic correlations can still develop even when there is directional sexual selection on sperm traits (*m*). Shown are the results of the first 50 generations of simulations when cryptic choice trait (*f*) starts 5 standard deviations higher than *m*. (A) Beta directional selection estimates of sperm trait over time (*m*; Lande & Arnold, 1983). Zero means no directional selection acting on sperm trait (black dashed line). (B) Genetic correlations between cryptic female choice trait and sperm trait. Dotted color lines indicated genetic correlations at generation 50 for the starting scenario in the main paper. Solid lines for both panels represent mean and bands represent standard deviation of 50 populations (separate runs) at each parameter combination.

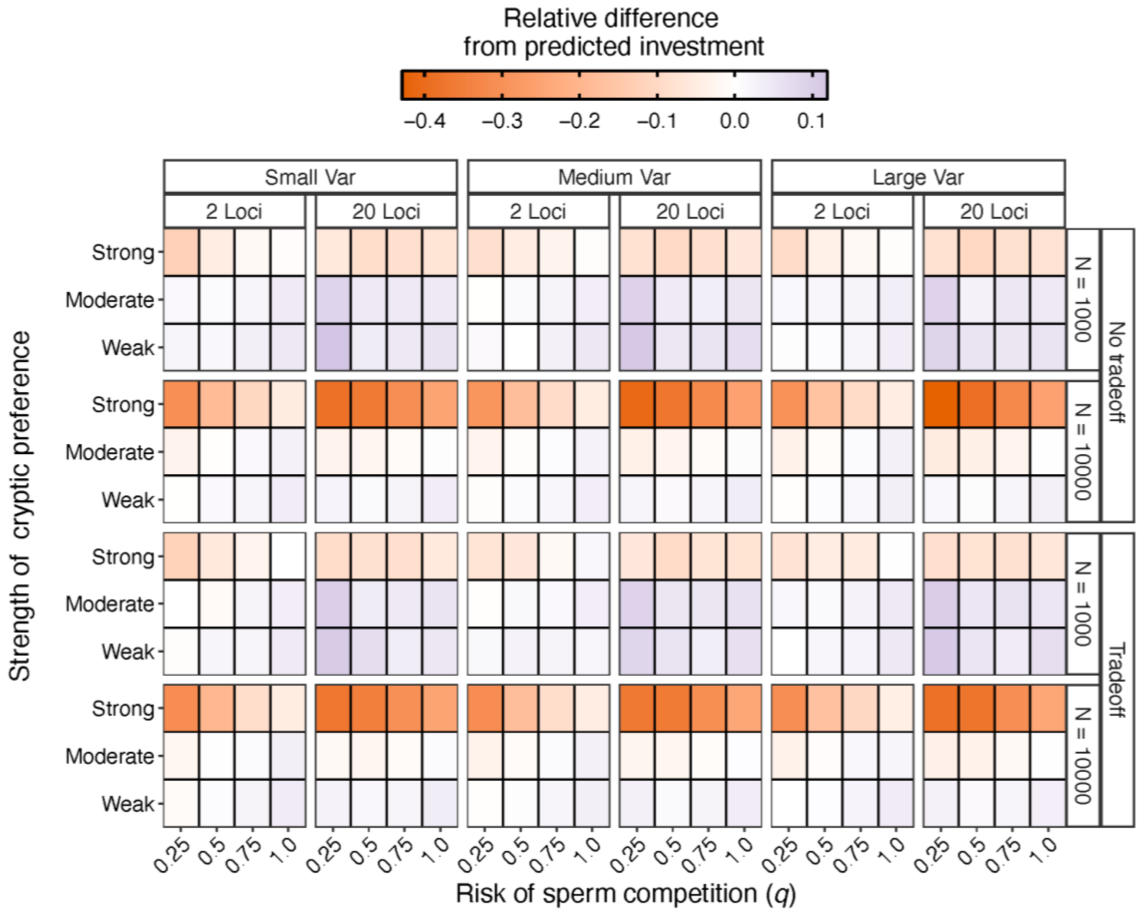

Fig. S8. Strong cryptic female choice results in less ejaculate investment than predicted by game theoretical models. Heat map of the median relative difference from predicted investment in ejaculates after 30,000 generations of evolution across different preference strengths, risks of sperm competition, population size, number of loci, starting variation, and whether a tradeoff between sperm number (*s*) and sperm trait (*m*) was present. Orange represents less investment than predicted by the analytical model, white means same as predicted, and purple means higher ejaculate investment than predicted. Ejaculate investment was calculated as *s* for no tradeoff scenario and *ms* for the tradeoff scenario. Results in the main text are from 20 Loci, medium variation, and population size of 10,000 (Fig. 2B).

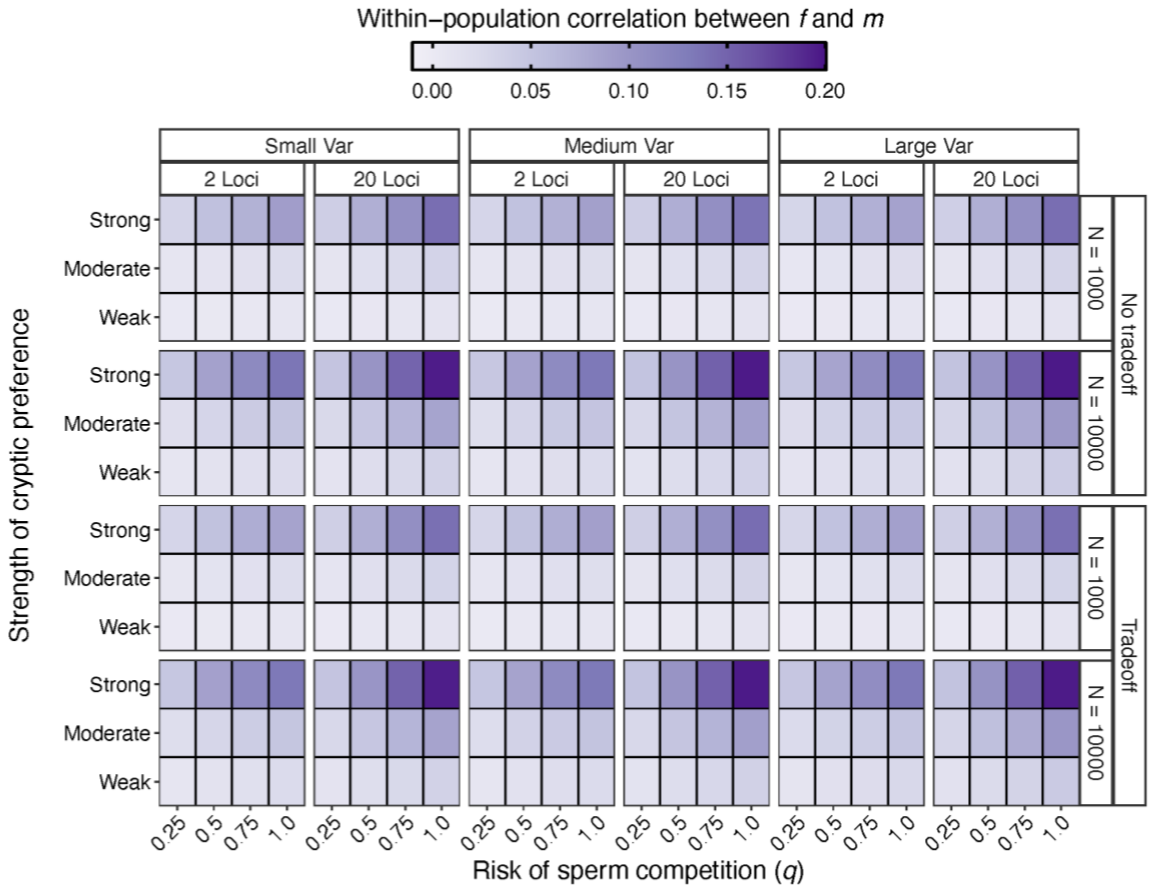

Fig. S9. Genetic correlations between cryptic female choice trait (*f*) and sperm trait (*m*) increase with risk of sperm competition and strength of selection. Heat map of the median within-population genetic correlation between *f* and *m* in the last 2,000 generations of evolution across different preference strengths, risks of sperm competition, population size, number of loci, starting variation, and whether a tradeoff between sperm number and sperm trait was present. The median was calculated as the median genetic correlation in the last 2,000 generations across 50 populations (runs) at each parameter combination. Results in the main text are from 20 Loci, medium variation, and population size of 10,000 (Fig. 3A).

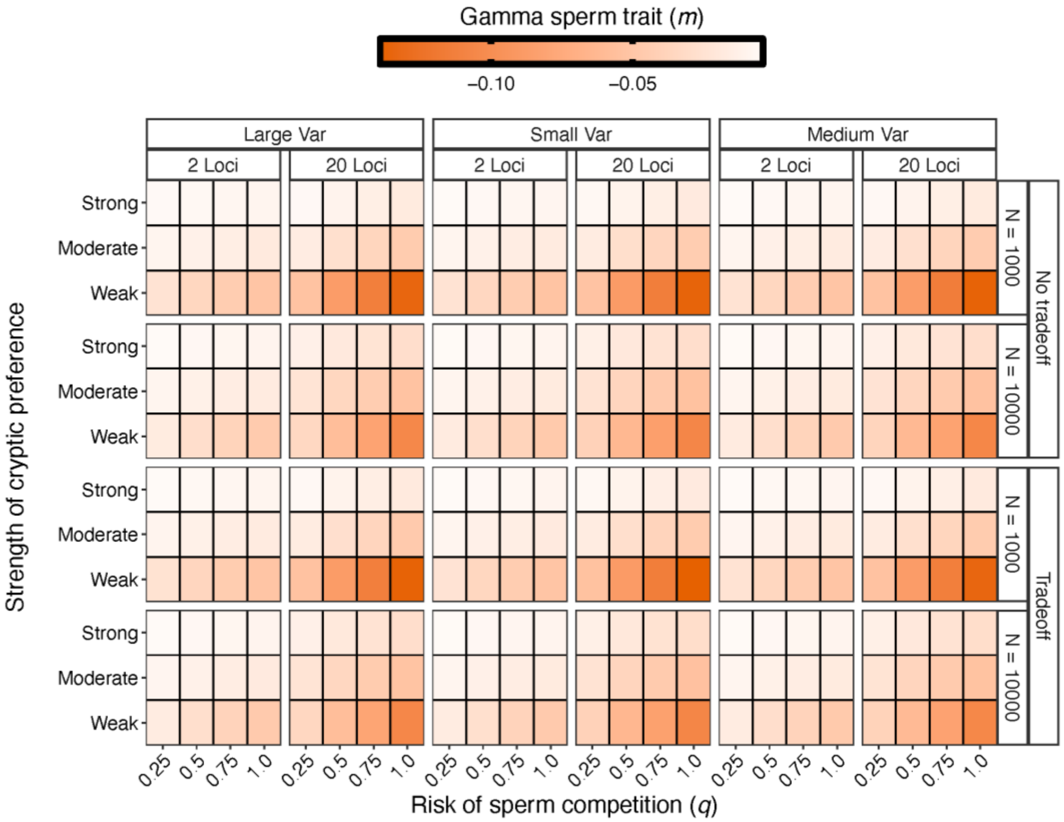

Fig.S10. Gamma selection estimates of sperm trait (*m*) decrease with increasing risk of sperm competition and strength of selection. Heat map of the median gamma selection estimate on *m* in the last 2,000 generations across different preference strengths, risks of sperm competition, population size, number of loci, starting variation, and whether a tradeoff between sperm number (*s*) and *m* was present. The median was calculated as the median gamma estimate on *m* in the last 2,000 generations across 50 populations (runs) at each parameter combination. Results in the main text are from 20 Loci, medium variation, and population size of 10,000 (Fig.4B).

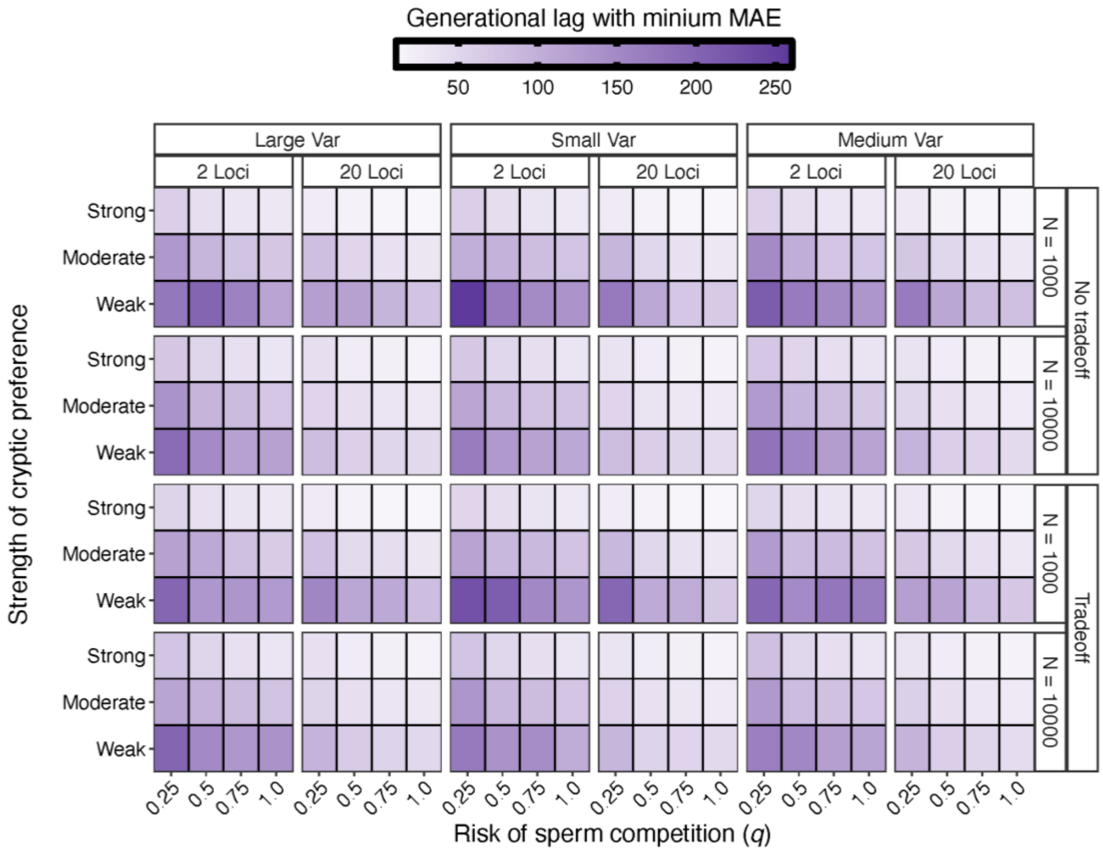

Fig. S11. Generational lag between sperm trait (*m*) and cryptic choice trait (*f*) decreases with increasing risk of sperm competition and strength of preference. Heat map of the median generational lag that minimized mean absolute error (MAE) between *f* and *m* in the last 2,000 generations across different preference strengths on *m*, risks of sperm competition, population size, number of loci, starting variation, and whether a tradeoff between sperm number (*s*) and sperm trait (*m*) was present. Results in the main text are from 20 Loci, medium variation, and population size of 10,000 (Fig. 5A).

Supplemental References

Lande, R. & Arnold, S.J. (1983) The Measurement of Selection on Correlated Characters. *Evolution*, **37**, 1210–1226.

Millan, C.H., Machado, G. & Muniz, D.G. (2020) Within-population variation in female mating preference affects the opportunity for sexual selection and the evolution of male traits, but things are not as simple as expected. *Journal of Evolutionary Biology*, **33**, 1579–1592.

Stinchcombe, J.R., Agrawal, A.F., Hohenlohe, P.A., Arnold, S.J. & Blows, M.W. (2008) ESTIMATING NONLINEAR SELECTION GRADIENTS USING QUADRATIC REGRESSION COEFFICIENTS: DOUBLE OR NOTHING? *Evolution*, **62**, 2435–2440.
